# Effects of a Self-Directed Clinical Practicum on Self-Confidence and Satisfaction with Clinical Practicum among South Korean Nursing Students: A Mixed-Methods Study

**DOI:** 10.3390/ijerph19095231

**Published:** 2022-04-25

**Authors:** Hyangjin Park, Haeryun Cho

**Affiliations:** 1 Department of Nursing, Catholic Kkottongnae University, Cheongju 28211, Korea; hjpark@kkot.ac.kr; 2 Department of Nursing, Wonkwang University, Iksan 54538, Korea

**Keywords:** clinical practicum, personal satisfaction, self-confidence, self-directed learning

## Abstract

In self-directed learning, students take the initiative to identify learning goals, choose learning strategies, and evaluate learning outcomes. This study aimed to explore the effects of a self-directed clinical practicum on nursing students’ self-confidence and satisfaction with the clinical practicum. This mixed-methods study used a non-equivalent control group with a pre- and post-test quasi-experimental design and content analysis. Participants were 111 nursing students (experimental group = 55, control group = 56). Quantitative (self-confidence and satisfaction with the clinical practicum) and qualitative (reflective diaries) data were collected. The experimental group had significantly increased scores for self-confidence and satisfaction with the clinical practicum compared with the control group. Four themes regarding the experience of the self-directed clinical practicum were identified: perceived linking of academic knowledge and practice, perceived development of nursing competency, enjoying the clinical practicum, and establishing nursing identity as a student. The self-directed clinical practicum developed as part of this study was found to be an effective education method for nursing students.

## 1. Introduction

The purpose of education in undergraduate nursing program is to prepare professional nurses through holistic classroom education, clinical practicum, and clinical simulation [1,2]. Particularly, clinical practicum is indispensable for nursing students to integrate nursing knowledge and clinical practice [3,4]. Effective clinical practicum programs may be a means to help nursing students increase their self-confidence, which could in turn increase their satisfaction with the clinical practicum [5].

Self-confidence reflects an individual’s beliefs in their skills and abilities [6]. In particular, effective clinical practicum was shown to improve nursing students’ knowledge, skills, and communication, consequently increasing their self-confidence [5,7]. An increase in self-confidence leads to better academic performance and nursing student’s satisfaction [5,8].

Nursing students’ high degree of satisfaction is related to their ability to perform in nursing practice [8,9]. Satisfaction with the practicum can influence clinical competency [3,8]. Additionally, nursing students’ satisfaction improves with effective practicum [5,9]. Therefore, nursing student confidence and satisfaction are important elements to consider in clinical practicum. 

The conventional clinical practicum provides unique learning opportunities for nursing students who are in direct contact with patients [4]. However, the clinical practice experience of nursing students may be passively limited by the increasing concerns for patient safety presented in real-world health care settings [10,11]. There is a great need for new clinical practicum education methods that overcome the limitation of conventional clinical practicum.

Nursing educators insist that nursing students must be self-directed, in order to become professional nurses [12,13]. Self-directed learning (SDL) was reported to improve not only academic performance but also knowledge, self-confidence, and satisfaction among nursing students [3,14,15]. Under SDL, nursing students take the initiative, with or without assistance from others, in identifying their learning needs [3,12]. They develop their learning goals, a process that includes identifying the resources for educational purposes, choosing and implementing suitable learning strategies, and evaluating learning outcomes [13,14,15]. Nursing education needs to develop an educational program that would help nursing students learn and explore nursing plans by themselves, thereby improving their self-confidence [13,15,16].

For these reasons, some nursing educators have applied SDL strategies to their programs for clinical simulation [7,8,9], basic nursing skills [5], and extra-curricular education [13]. SDL was found to be effective in improving the knowledge, self-confidence, and satisfaction of nursing students [5,7,8,9,13]. However, because of the lack of studies on this educational strategy applied in the clinical setting, it is necessary to check whether SDL is effective in clinical practicum. Moreover, the literature primarily includes descriptive or experimental studies, not mixed-method design. Therefore, the present study used a sample of nursing students and explored the effects on self-confidence and satisfaction of applying the SDL with a mixed method proposed by Garrison [17] to a clinical practicum program.

## 2. Background

**Figure 1:**
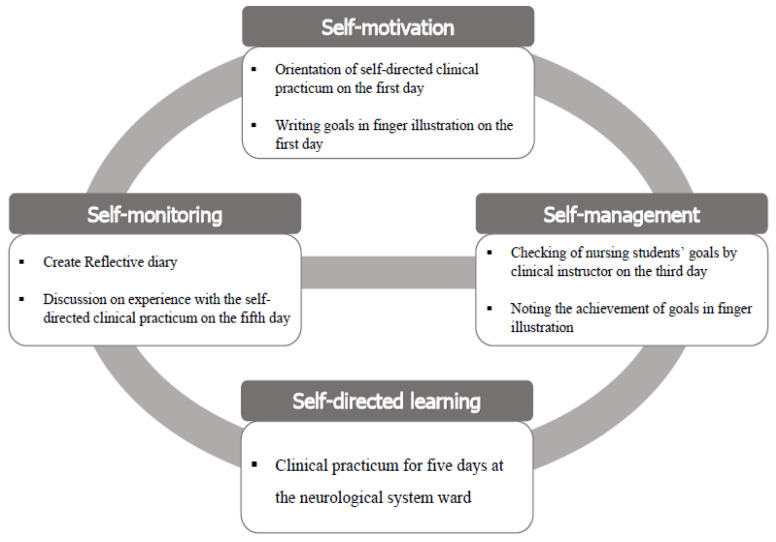
Conceptual framework.

Garrison’s SDL model [17] was used as the conceptual framework for the verification of the effects of the self-directed clinical practicum (SDCP) in this study (Figure 1). This model is reportedly characterized by its strength in establishing interesting interactions between the instructor and the student, improving academic achievement and quality, and promoting the achievement of learning outcomes [15]. The SDL model includes three major concepts: self-motivation, self-management, and self-monitoring [17].

Self-motivation involves the initiation and maintenance of efforts toward learning and achieving cognitive goals [17], as well as the learning of a task and then taking responsibility for planning one’s own learning outcomes and activities [3,15]. In this study, a clinical instructor provided an orientation to SDCP to nursing students, and then the students planned their individual goals using illustrations.

Self-management refers to focusing on the management of available learning resources and support [17]. The term “resources” in this context indicates the materials and feedback on student learning activities [3,15]. In this study, a clinical instructor provided feedback and various resources relevant to the nursing students’ goals, and the nursing students checked the achievement of their own goals using illustrations. 

Self-monitoring refers to the conscious monitoring of one’s own learning, thinking, and actions through self-evaluation, self-reflection, and metacognition [17]. In this study, nursing students maintained a reflective diary on their experiences and self-evaluation of the SDCP. They discussed their experience with the SDCP and their learning and thinking through the reflective diary.

## 3. Purpose

This study aimed to explore the effects of an SDCP for Korean nursing students on their self-confidence and satisfaction with the clinical practicum.

## 4. Materials and Methods

### 4.1. Design

A mixed-methods study was conducted to evaluate the effects of the SDCP. A non-equivalent control group with a pre- and post-test quasi-experimental design was used for the quantitative study. A content analysis design was used for the qualitative study.

### 4.2. Setting

This study was carried out at two universities in Jeolla-do, South Korea. The two universities were the same in terms of curricula, size, and type (i.e., 4-year university). Both were certified by Korean Accreditation Board of Nursing Education. The two universities were similar with respect to their clinical practicum curriculum. During the first semester of the fourth year, nursing students attend an adult nursing practicum at the university hospital for 5 days. Therefore, the two universities were considered to follow a similar curriculum, as well as have the same setting and characteristics of students. To avoid spreading information about the study, the researchers selected two hospitals that were approximately 100 km apart. Hence, the participants in one hospital had limited opportunities to interact with those in the other hospital.

### 4.3. Sample

The participants in this study were recruited from senior grade in nursing college. The senior grade students from both schools were completing an adult nursing practicum in which they cared for patients with neurological problems. The inclusion criteria for participants were as follows: (1) completion of two or more semesters of conventional clinical practicum; (2) enrollment in an adult nursing practicum with similar curriculum in the two universities to ensure internal validity; and (3) enrolment in a clinical practicum in a department of the neurology ward, neurosurgery ward, neurosurgery intensive care unit, or stroke center at the university hospitals. The exclusion criteria were as follows: (1) did not participate in the clinical practicum during weekdays owing to holidays or absence, or (2) had previous experience with SDL.

The study used G*Power software version 3.1.7 (Heinrich Heine Universitat, Dusseldorf, Germany) [18] to determine the required number of participants per group (51), in order to achieve a medium effect size with an alpha of 0.05 and power of 0.80. Considering the expected dropout rate, 60 participants were included in each group. At the end of the program, five nursing students in the experimental group withdrew (two refused to participate further and three had incomplete responses); four nursing students in the control group withdrew, stating “refusal to continue” as the reason. Overall, the study had 111 participants (55 in the experimental and 56 in the control group).

### 4.4. Measurement

#### 4.4.1. Self-Confidence

A 10-item self-report scale to assess self-confidence in clinical settings was developed by the authors in this study (Appendix A). The scale measured clinical confidence in solving patients’ neurological problems. Some of the areas of confidence included confidence in areas of nursing processes, such as nursing assessment, diagnosis, plans, practice, and evaluation. Each item was measured on a five-point Likert-type scale from 1 (completely disagree) to 5 (completely agree), with higher scores indicating higher self-confidence. The self-confidence score was calculated by average values of the 10 items, the score range was from one to five. Cronbach’s alpha was 0.91, indicating good reliability.

#### 4.4.2. Satisfaction with the Clinical Practicum

A seven-item self-report scale to measure satisfaction with the clinical practicum was developed by the authors (Appendix A). Each item was measured on a five-point Likert scale ranging from 1 (completely disagree) to 5 (completely agree), with higher scores indicating higher satisfaction. The satisfaction with the clinical practicum score was calculated by average values of the seven-items the score range was from one to five. Cronbach’s alpha was 0.71, indicating suitable reliability.

#### 4.4.3. Content Validity

The content validity of the instruments used in this study was reviewed by five professors teaching at nursing colleges, with doctorates and more than 4 years of teaching experience. At the time of the study, they taught at a college different from where this study was conducted to prevent bias. They evaluated the suitability of the items and confirmed the items’ appropriateness to assess clinical confidence and satisfaction with the clinical practicum. Each expert assessed the appropriateness of each item and variable using a two-point Likert scale: 0 (not valid at all) and 1 (valid). The content validity index of the tools used in the current study was above 0.95, indicating high validity.

### 4.5. Self-Directed Clinical Practicum

**Figure 2:**
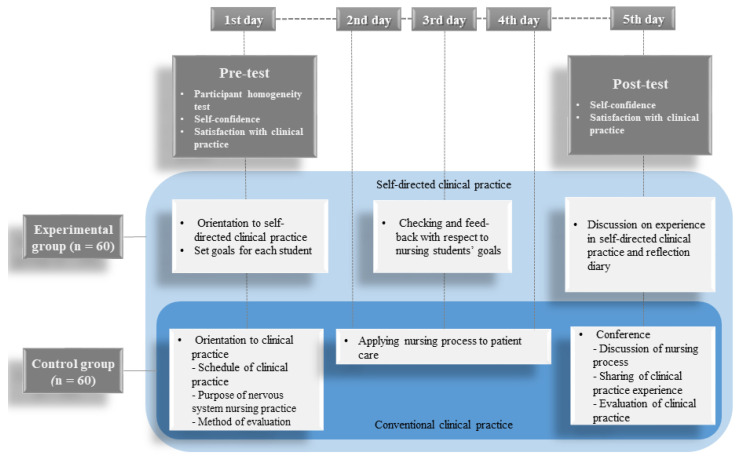
Study process.

Researchers in this study developed an SDCP program that involved the care of patients with neurological problems for 5 days (Figure 2). The conventional clinical practicum program was provided to both groups. The details of conventional clinical practicum are as follows. On the first day of the clinical practicum, the clinical instructor provided the general guidelines for neurological practice, such as the schedule of clinical practicum and the purpose and method of evaluation of the neurological nursing practicum. The aim of the neurology nursing practicum was to provide nursing students with critical thinking capabilities in scientific, systematic, and comprehensive nursing care to address patients’ neurological problems. From the second to the fourth days of the clinical practicum, nursing students applied the nursing process to their patients, whom they selected based on the latter’s neurological issues. The clinical instructor checked the nursing process and provided feedback to the nursing students. On the fifth day, the clinical instructor and nursing students discussed the nursing process and shared their clinical practice experience in a conference-type session. 

**Figure 3:**
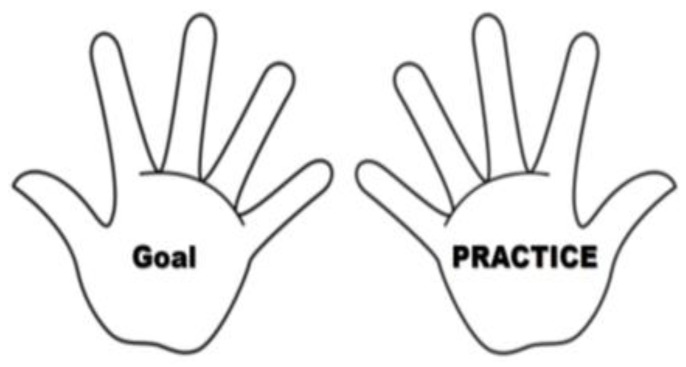
Sample of finger illustration.

The SDCP program was added for the experimental group. On the first day of the clinical practicum, the content of the SDCP program was explained in detail by the clinical instructor. Each nursing student set goals for his/her clinical nursing practicum with neurology patients. To visualize the clinical practicum goals in detail, the nursing students were instructed to use an illustration of two hands designed by the study authors; the illustration helped students organize the goals and attainment process that they set for themselves and succeed in SDCP. The nursing students were asked to write their clinical practice goals on the fingers of the left hand, and the contents of clinical performance on the fingers of the right hand (Figure 3). Subsequently, a list of nursing practices relevant to the neurological care setting was provided to nursing students to aid in goal-setting. This list included information on the assessment of loss of consciousness (LOC), assessment of motor grades, physical therapy, and other nursing skills. 

On the second day of the clinical practicum, the nursing students conducted their clinical practice to achieve their practice goals in each clinical field. On the third day, the clinical instructor checked how each nursing student achieved their own goals, and then provided support and encouragement, such as reference to related useful information, books, and registered nurses (RNs) who may be able to help. The nursing students modified their practicum goals or plans based on the clinical instructor’s detailed feedback. An example is a student’s change in plans from “conducting nursing procedures as a skillful registered nurse” to “learning how to perform basic nursing skills correctly”. Another student had a plan to “assess LOC five times a day”, which was changed to “assess LOC precisely regardless of the number of times”. In this case, the assessment results were compared with the LOC assessment results of practicing nurses. If differences were seen between the student and nurse assessments, the clinical instructor recommended that the student revise his/her incorrect assessment. 

On the fourth day, students applied the modified goals to their clinical practicum. On the final day of the clinical practicum, the clinical instructor and nursing students shared and discussed their achievement results and clinical practicum experiences. In addition, the nursing students submitted their reflective diary to the clinical instructor, with daily entries regarding their experience with the SDCP and self-evaluation.

### 4.6. Data Collection

Data were collected from 3 April to 16 June 2017. On the first day of the clinical practicum, the researchers collected the participants’ general characteristics and conducted pre-tests. During the learning period, the same number of clinical instructors and registered nurses were applied to both groups. The post-tests were performed on the fifth day of the practicum. The self-confidence and satisfaction with the clinical practicum questionnaires were collected in person; they took an average of 5 minutes for participants to complete. After the clinical practice was over, the reflection diaries were collected from the experimental group to collect qualitative data.

### 4.7. Ethical Considerations

This study was approved by the Institutional Review Board of W University (IRB no. WKIRB-201703-SB-017). Before clinical practicum, the authors explained the purpose and contents of this study to the participating nursing students, highlighting that participation versus non-participation carried no advantages. Informed consent was obtained from all the nursing students who voluntarily decided to participate in this study. To avoid the Hawthorne and halo effects, the data was obtained by clinical instructors who were not in this research group.

### 4.8. Data Analysis

Quantitative data analyses were completed using SPSS 24.0 (IBM Inc., Chicago, IL, USA). Cronbach’s alpha was used to evaluate internal consistency. Chi-squared and *t*-tests were performed to examine the homogeneity of the experimental and control groups. As a result of checking the skewness and kurtosis of the collected data, it was found to be within ±1.97. Therefore, the assumption of normality distribution was satisfied [19]. An independent-samples *t*-test was run to assess the difference in pre- and post-test scores between the two groups. Effect size was calculated by Cohen’s d formula.

Krippendorff’s [20] content analysis was used to analyze the qualitative data. First, the researchers repeatedly read the reflective diaries and identified the contents related to the SDCP. Second, among these contents, meaningful statements were extracted, and meanings were conceptualized. Third, similar or related concepts were grouped.

## 5. Results

### 5.1. Homogeneity Test

Table 1 presents the results of the homogeneity test between the experimental and control groups before the intervention. No significant differences in any of the participants’ general characteristics were found between the two groups. The nursing students in the two groups were similar in terms of gender (χ^2^ = 0.07, *p* = 0.794), age (*t* = 0.56, *p* = 0.577), perceived academic grade (χ^2^ = 4.63, *p* > 0.999), and general satisfaction with the clinical practicum (*t* = 0.19, *p* = 0.854). There were no significant differences in the baseline score for the dependent variables between the two groups. The participants in the two groups were similar with regard to self-confidence (*t* = 0.80, *p* = 0.427) and satisfaction with the clinical practicum (*t* = 1.24, *p* = 0.217), and the two groups were thus considered homogenous.

### 5.2. Effects of Self-Directed Clinical Practicum

Table 2 presents the *t*-test results of the pre-post-score difference between the two groups in self-confidence and satisfaction with the clinical practicum. The experimental group showed a significantly more increased score for self-confidence (*t* = 3.98, d = 0.76, *p* < 0.001) and satisfaction with the clinical practicum, compared with the control group (*t* = 6.53, d = 1.25, *p* < 0.001).

### 5.3. Experience of Self-Directed Clinical Practicum

A total of 102 meaningful statements were extracted from the reflective diaries. They were categorized into four themes: perceived linking of academic knowledge and practice, perceived development of nursing competency, enjoying the clinical practicum, and establishing nursing identity as a student.

#### 5.3.1. Perceived Linking of Academic Knowledge and Practice

The nursing students made efforts to care for patients with neurological problems using the academic knowledge they gained in school. Unlike in the conventional clinical practicum, these efforts enabled them to apply their academic knowledge to the clinical practicum more actively. The following are statements from the nursing students’ diaries (translated by the authors).

“So far, I have had clinical practicum experiences that separated academic knowledge from the clinical practicum. In this neurology clinical practicum, as I tried to define my nursing plans and goals, I applied my knowledge naturally to my practicum as much as possible. Consequently, I got satisfactory results, and I was very satisfied with this practicum”.

“There was a clear difference between what I learned in theoretical classes in school and what I did in clinical practice. I think learning something by doing it in person during clinical practice made it clearer and easier to understand”.

#### 5.3.2. Perceived Development of Nursing Competency

Instead of passively following the goals or tasks that were assigned at school or in the clinical practicum, the nursing students could practice nursing skills or develop knowledge that they chose, and this allowed them to define and create their own path of development. The following are statements from the nursing students’ diaries (translated by the authors).

“It was an opportunity to become more active in the clinical practicum, and I saw development in my nursing knowledge and skills as I revised and changed my unsatisfactory areas. I also experienced self-reflection”.

“The more I practiced, the more questions I had; this urged me to practice more. Through this, I found my own development, unlike in my previous clinical practice”.

#### 5.3.3. Enjoying the Clinical Practicum

As the nursing students chose the contents of the clinical practicum themselves, they realized the breadth of the clinical field. Consequently, these experiences increased their interest and active participation in the clinical practicum. The following are statements from the nursing students’ diaries (translated by the authors).

“I was really worried about the practicum in adult nursing, as I expected it to be the most difficult part, but contrary to my expectation, I found it to be so enjoyable. I enjoyed this practicum”.

“I became more interested in nursing while doing SDCP. Unlike before, I tried to learn and tried hard. I thought the RNs liked my behavior and answered my questions more kindly”.

“This practicum was so nice and beneficial for me, because I set up the nursing goals or skills that I wanted to attain, and I tried to practice one every day. Therefore, I never felt it was very difficult, and it made me excited to practice more”.

#### 5.3.4. Establishing Nursing Identity as a Student

Owing to the SDL component, the nursing students became more involved and active in the practicum experience. They also had an opportunity to deeply consider the question, “What is nursing?” In this way, the SDCP helped them establish their nursing identity as student nurses. The following are statements from the nursing students’ diaries (translated by the authors).

“I was able to observe and practice more comprehensive nursing care rather than checking electronic nursing records for my homework, and I felt that a nurse’s job was very appealing”.

“It was a clinical practicum that allowed me to think deeply about nursing”.

## 6. Discussion

Nursing students that received the SDCP showed a significantly higher increase in self-confidence in clinical settings compared with the control group students. Moreover, according to Cohen’s criterion [21], the effect size of SDCP on self-confidence was large. Similarly, Lee and Shin [5] suggested that self-directed feedback practice was effective in improving self- confidence in basic nursing skills. Several studies have also reported that the most effective factor in increasing self-confidence in nursing students was SDL ability, with higher SDL ability being associated with higher self-confidence [16,22]. Shahrouri [15] stated that SDL allows students to take responsibility and control their learning to be meaningful. Therefore, the increase in self-confidence could be attributed to their self-control in making clinical practicum meaningful through SDCP. 

Further, some studies have found that SDL leads to higher levels of academic knowledge and interest [14,15,23]. In the qualitative analysis, the nursing students in the current study indicated that because they chose the knowledge and skills that they wanted to develop, it was easier to remember this information than it would have been if they learned from a textbook alone. This result suggests that SDL could be an effective way to precisely determine nursing students’ clinical practicum goals and successfully connect the clinical practicum with academic knowledge. Additionally, this experience could improve the self-confidence and nursing competence of nursing students. The large effect size of SDCP on self-confidence, verified in this study, supports this implication. Therefore, as self-direction is an essential factor in the clinical practicum of nursing students, researchers must develop more varied educational methods involving SDL in the clinical practicum.

Meanwhile, the nursing students reported that owing to their attitude, including enthusiasm and activeness, they eventually developed a positive relationship with RNs in the hospital. In addition, they had an opportunity to consider the influences of nursing and professionalism. Nursing students in the experimental group showed a significantly higher increase in satisfaction with the clinical practicum, compared with those in the control group. The effect size of SDCP on satisfaction with clinical practicum was large [21]. These results suggest that nursing students experienced a good model in the clinical setting and formed a nursing profession identity. Thus, they were able to enjoy the clinical practicum. Several studies have reported that the most influential factor on satisfaction with a practicum was SDL ability [5,8,16]. It was considered that the effect size of SDCP was large [21] on satisfaction with clinical practicum because of this background.

However, one study reported nursing students being dissatisfied with SDL in clinical settings [3]. Identifying resources for learning and basic self-management ability can have the greatest influence on satisfaction with the clinical practicum, and could result in dissatisfaction with SDL [3,16]. It can be difficult to find resources in classroom and clinical settings, which may lead to a decrease in students’ satisfaction with the clinical practicum [12,14]. For these reasons, in the present study, clinical instructors checked the degree of achievement of each nursing student’s clinical practicum goals and ensured that the students could obtain the resources needed to achieve them. In turn, SDL enhanced the students’ motivation to improve their efforts [15]. The clinical instructors also provided information on books with useful content and information regarding ways to obtain help from RNs in the ward during clinical practicum. Therefore, satisfaction with the clinical practicum of the experimental group showed a higher increase, compared with the control group. These results suggest that SDL may be considered effective for enhancing satisfaction with the clinical practicum.

The current study adopted the SDL model as a conceptual framework [17]. This study provided an opportunity for nursing students to experience an SDL method and evaluate its effect when applied to a clinical practicum. In the conventional clinical practicum, clinical instructors usually set the learning goals, and nursing students attend sessions passively. In SDCP, nursing students set detailed practice goals themselves by identifying their areas of interest or deficiency. Then, clinical instructors help the nursing students achieve their goals. The findings showed that SDCP was an effective educational method to improve the self-confidence and satisfaction with the clinical practicum in nursing students. For nursing students, the SDCP provided a valuable experience to connect their nursing knowledge with the clinical field and allowed them to experience self-development by establishing their nursing identity. Consequently, the SDCP could be an effective strategy for nursing education, based on the interactions between clinical instructors and nursing students. 

The clinical practicum is the core component in undergraduate nursing program [2,4]. As such, an important contribution of this study is the development of an SDL-based clinical practicum. Moreover, the comprehensive analysis of quantitative and qualitative data to examine the effects of the SDCP provides strong evidence. However, participants in this study were not chosen by random sampling, as there was non-randomized assignment between groups. Additionally, SDCP was applied only to neurological nursing care in a clinical practicum. These limitations may limit the generalizability of the results.

## 7. Conclusions

An important contribution of the current study is the finding that an SDL-based clinical practicum led to a higher improvement in nursing students’ self-confidence and satisfaction with the clinical practicum, compared with a conventional program. Therefore, the SDCP may be an effective pedagogical method in the education of nursing students. 

Based on these results, this study provides the following suggestions. The SDCP need to be applied, validated and evaluated not only in adult nursing but also in other areas, such as pediatric, mental health, maternal, and community health nursing. Additionally, educational programs using SDL need to be developed and evaluated for other grades besides the senior grade in nursing college.

## Figures and Tables

**Table 1:** Homogeneity test between two groups (*n* = 111).

Characteristics	Classification	Exp. (*n* = 55)	Con. (*n* = 56)	χ^2^/t	*p*
		*n* (%)/M ± SD	*n* (%)/M ± SD		
General characteristics					
Gender	Male	6 (10.9)	7 (12.5)	0.07	0.794
	Female	49 (89.1)	49 (87.5)		
Age		21.86 ± 1.20	21.98 ± 1.15	0.56	0.577
Perceived academic grade	High	2 (3.6)	6 (10.7)	4.45 *	0.126
	Moderate	49 (89.1)	41 (73.2)		
	Low	4 (7.3)	9 (16.1)		
General satisfaction of clinical practicum		3.25 ± 0.63	3.25 ± 0.65	0.19	0.854
Completed clinical practicum subjects	Adult nursing	55 (100.0)	56 (100.0)	-	-
	Psychiatric nursing	54 (98.2)	54 (96.4)	0.33 *	>0.999
	Pediatric nursing	54 (98.2)	56 (100.0)	1.41 *	0.495
	Woman’s nursing	54 (98.2)	55 (98.2)	0.00 *	>0.999
	Community nursing	20 (36.4)	56 (100.0)	66.27 *	<0.001
	Nursing management	35 (63.6)	42 (75.0)	1.69	0.194
Baseline score					
Self-confidence		3.26 ± 0.43	3.33 ± 0.59	0.80	0.427
Satisfaction with the clinical practicum		3.21 ± 0.40	3.32 ± 0.47	1.24	0.217

Con. = control group, Exp. = experimental group, M ± SD = mean ± standard deviation. * Fisher’s exact test.

**Table 2:** Effects of self-directed clinical practicum (*n* = 111).

Variable	Experimental Group (*n* = 55)			Control Group (*n* = 56)			t	E.S.	*p*
	Mean ± SD			Mean ± SD					
	Baseline Score	Final Score	Change Score	Baseline Score	Final Score	Change Score			
Self-confidence									
	3.26 ± 0.43	3.71 ± 0.56	0.46 ± 0.44	3.33 ± 0.59	3.46 ± 0.54	0.13 ± 0.44	3.98	0.76	<0.001
Satisfaction with the clinical practicum									
	3.21 ± 0.40	3.85 ± 0.38	0.64 ± 0.40	3.32 ± 0.47	3.48 ± 0.46	0.17 ± 0.35	6.53	1.25	<0.001

Change score = Final score—Baseline score, SD = standard deviation; E.S. = Effect size.

## Data Availability

The data presented in this study are available on request from the corresponding author.

## Appendix A. The List of Questions

### Appendix A.1. Self-Confidence

1. I am confident in collecting patient’s health history data.
2. I am confident in performing a physical assessment.
3. I am confident in analyzing meaningful data.
4. I am confident in making a nursing plan according to the nursing diagnosis.
5. I am confident in making nursing diagnoses according to nursing problems.
6. I am confident in changing the nursing plan according to the change of the patient’s condition.
7. I am confident in planning care that meets the needs of patients and their families.
8. I am confident in making a nursing plan that can be combined with the doctor’s treatment plan.
9. I am confident to include the use of community resources in nursing plans for patients and their families.
10. I am confident in understanding the basic principles of a doctor’s prescription.

### Appendix A.2. Satisfaction with the Clinical Practicum

1. Clinical practicum provided new experiences according to the learning progress.
2. Rather than simply learning, it was practiced to experience actual nursing work.
3. What I learned at school was also applied to clinical practice.
4. During clinical practice, I took care of patients according to the nursing process.
5. The practice was focused on applying the knowledge learned during the lecture.
6. I performed problem-focused nursing on patients rather than simple functional tasks.
7. I sometimes had nothing to do during clinical practice.
